# Identification of Hypsarrhythmia in Children with Microcephaly Infected by Zika Virus

**DOI:** 10.3390/e21030232

**Published:** 2019-02-28

**Authors:** Gean Carlos Sousa, Claudio M. Queiroz, Patrícia Sousa, Priscila Lima, Antônio Augusto Moura da Silva, Nilviane Pires, Allan Kardec Barros

**Affiliations:** 1 Department of Electrical Engineering, Federal University of Maranhão (UFMA), São Luís-MA 65080-805, Brazil; 2 Brain Institute, Federal University of Rio Grande do Norte, Natal 59078-970, Brazil; 3 Department of Medicine, University Hospital of the Federal University of Maranhão, São Luís-MA 65080-805, Brazil; 4 Department of Public Health, Federal University of Maranhão, São Luís-MA 65080-805, Brasil; 5 Program in Biotechnology, Center for Biological Sciences and Health, Federal University of Maranhão (UFMA), São Luís-MA 65080-805, Brazil

**Keywords:** hypsarrhythmia, electroencephalographic, epileptic, identification, index

## Abstract

Hypsarrhythmia is an electroencephalographic pattern specific to some epileptic syndromes that affect children under one year of age. The identification of this pattern, in some cases, causes disagreements between experts, which is worrisome since an inaccurate diagnosis can bring complications to the infant. Despite the difficulties in visually identifying hypsarrhythmia, options of computerized assistance are scarce. Aiming to collaborate with the recognition of this electropathological pattern, we propose in this paper a mathematical index that can help electroencephalography experts to identify hypsarrhythmia. We performed hypothesis tests that indicated significant differences in the groups under analysis, where the *p*-values were found to be extremely small.

## 1. Introduction

The Congenital Zika Virus Infection can cause severe brain abnormalities and diverse electroencephalographic abnormalities [1]. Among these is hypsarrhythmia, an unusual activity of the electroencephalogram (EEG), found in a few types of epilepsy and known for interictal and spike-and-wave discharges in an irregular and disorganized background [2,3,4,5,6].

Identifying hypsarrhythmia patterns is challenging, and only experienced professionals in the reading of EEG tend to identify the presence of hypsarrhythmia distinctly [4,7,8]. This task tends to be even harder when the analysis is made in EEG exams of nursing infants infected by the Zika virus and with microcephaly, because, as is pointed out by Carvalho et al. (2017), these exams are consistently abnormal even in infants who have not yet developed epilepsy [1]. Additionally, brief and subtle seizures may not be detected, or can cause diagnostic errors [4].

Carvalho et al. (2017) found hypsarrhythmia in EEGs of newborns infected by the Zika Virus and with microcephaly [1]. The presence of hypsarrhythmia in EEGs in the sleep of children with microcephaly was also found by Kanda et al. (2018) [9]. It is worth mentioning that these works performed only the visual description of EEG signals due to the lack of mathematical and computational methods that can assist experts carrying out these types of tasks.

According to Roger (2015), the conventional systems of classification do not take into account all the ramifications of the impact of diagnostic errors [10]. For this reason, we propose a mathematical index capable of assisting the identification of hypsarrhythmia in a fast and efficient way. The mathematical features of this index are grounded on the morphological similarities that exist between a spike and a Gabor function. The main hypothesis for the definition of the index is that the inner product between a Gabor function and the parts of the signal that contain spikes always presents higher values than the products between the same Gabor and the rest of the signal.

The proposed methodology tends to provide evidence for the differences between electroencephalograms with a hypsarrhythmia pattern and normal electroencephalograms with normal tracings, because according to Mytinger et al. (2018), electroencephalographic tracings with hypsarrhythmia have a greater number of spikes than the normal tracings [11].

## 2. Materials and Methods

### 2.1. Database

The database used was composed by EEGs of 30 children with the Congenital Zika Syndrome, with a total of 203 EEGs of 5 minutes each (98 with traces of hypsarrhythmia and 105 without any abnormalities). These signals were obtained by the government institution “Casa Ninar” [12], which offers medical assistance to children with microcephaly. The children assisted in the institution have different electroencephalographic abnormalities and, in some cases, have epileptic crises. Clinically, these crises are marked by infantile spasms linked with the presence of hypsarrhythmia. The electroencephalographic records were collected with electrodes positioned in accordance with the 10–20 system during periods of spontaneous sleep. The software Neuromap was used to collect data, and the signals were sampled at 128 HZ. An expert identified the presence or absence of hypsarrhythmia on the EEGs. Consent for the use of the collected data was obtained by means of the Ethics Committee of the Federal University of Maranhão, under the registration code CAAE 65897317.1.0000.5086.

### 2.2. Hypsarrhythmia Index

A large number of spikes in an electroencephalographic record can be characterized as a hypsarrhythmic record [7]. Spikes are electrographic markers where its quantification is used in the surgical planning of patients with epilepsy. A spike is clinically defined as a transitory acute that lasts between 20 and 70 ms and that is clearly distinguishable from background activity [13,14,15,16].

The index proposed in this paper is based on an inner product between an EEG and a Gabor function, by means of the discrete wavelet transform. A Gabor function is a sinusoidal function modulated by a Gaussian function [17,18,19,20] (1).

(1) $$\varphi \left(t\right)=Ae^{-\frac{{\left(t-\xi \right)}^{2}}{2\sigma^{2}}}\cos\left[\left(t-\xi \right)\omega +\phi \right]$$

The parameters ξ,σ,ω, and ϕ are, respectively, the average and the standard deviation of the Gaussian envelope, the frequency, and the cosine phase. By varying the parameters, it is possible to achieve an infinity of waves.

The Discrete Wavelet Transform (DWT) is the inner product between a signal $X_{n}$ and a Wavelet $\psi_{a,b}\left(n-b\right)$, moving $\psi_{a,b}\left(n-b\right)$ through the parameter *b* [21,22]. The DWT is given as follows (2):

(2) $$DWT=\sum_{n=1}^{N}X_{n}\psi_{a,b}\left(n-b\right).$$

**Figure 1:**
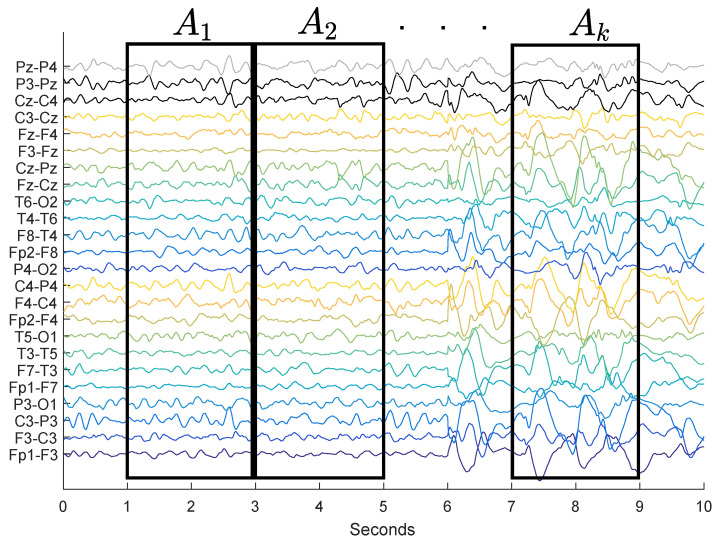
Windowing of the EEG’s signal in $A_{k}$ matrices.

The function $\psi_{a,b}\left(n-b\right)$ used in our experiments will be the Gabor function. The Gabor functions were chosen as the core of the DWT, since they are morphologically similar to the waves known as spikes. The DWT will be applied in windows of *w* seconds of EEG and after that, the energy of the coefficients of this transformation will be analyzed. Each window assessed corresponds mathematically to a Matrix $A_{k}$ of dimensions m×n, in which *m* is the number of channels and *n* is the number of samples (Figure 1).

**Definition** **1** (Hypsarrhythmia Index)**.** *Given that $\alpha_{k}=\left\{\alpha_{i1},\alpha_{i2},\alpha_{i3},\ldots ,\alpha_{in}\right\}$ the set of coefficients of the DWT of the i-th matrix line $A_{k}(m$ x n). We defined the hypsarrhythmia index as follows (3):*

(3) $$I_{k}=\frac{\sum_{i=1}^{m}\sum_{j=1}^{n}\left|\alpha_{ij}\right|}{m\;n}$$

Thus, for each window of *w* seconds of the EEG signal, we get an $I_{k}$ index, and consequently, for a signal of *K*-windows, we get a $\beta =\{I_{1},I_{2},I_{3},\ldots ,I_{K}\}$ sequence.

### 2.3. Feature Extraction and Classification

The process of feature extraction proposed in this paper was accomplished in three stages. First, the EEG went through a signal windowing (Figure 1). Afterward, the hypsarrhythmia index was calculated in each window, thus obtaining a β sequence of indexes. Finally, the average and the log energy entropy of the sequence were calculated. The log energy entropy is used to assess the set of indexes of a signal, and is defined as follows [23,24,25]:

(4) $$E_{LgEn}=\sum_{i=1}^{N}\log\left(x_{i}^{2}\right),$$

**Figure 2:**
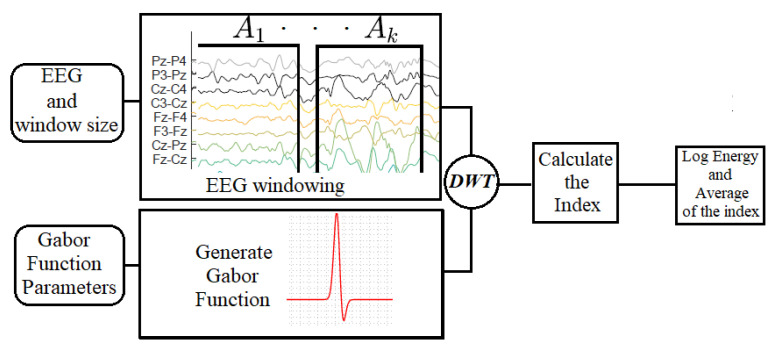
Algorithm for feature extraction.

In which $x_{i}$ is the *i*-th signal sample of length *N*. The sequence of indexes $\beta =\{I_{1},I_{2},I_{3},\ldots ,I_{K}\}$ from an EEG signal to compute the log energy entropy (4). A summary of the feature extraction process can be seen in Figure 2.

## 3. Results

In our simulations, we used 203 electroencephalogram signals of children with microcephaly. These EEGs had a length of 5 min each, and were segmented in 300 windows of 10 s. We performed experiments with windows of 3 and 20 s, and we observed that there were not any significant differences in the results. For each window, an index $I_{k}$ was calculated in Equation (3), composing a sequence of 300 indexes for each EEG. The average μ and the log energy entropy $E_{LgEn}$ of each sequence were calculated, considering different parameter combinations of the Gabor functions.

**Figure 3:**
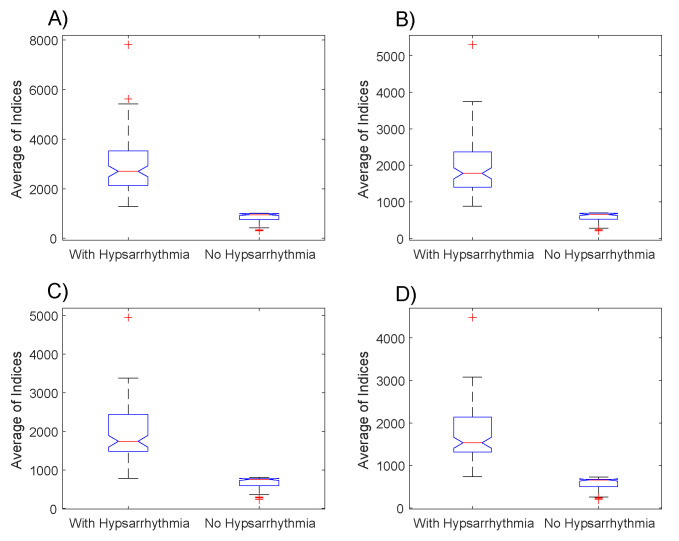
Boxplot with the averages of the EEGs’ indexes with and without hypsarrhythmia. Indexes generated from the Gabor function with the parameters: (**A**) $\sigma^{2}=20$, ω=1.02 and ϕ=5, (**B**) $\sigma^{2}=5$, ω=1.40 and ϕ=5, (**C**) $\sigma^{2}=15$, ω=2.68 and ϕ=5, (**D**) $\sigma^{2}=10$, ω=3.20 and ϕ=5.

Many computational experiments were made in order to identify the EEGs with hypsarrhythmic patterns. The boxplot presented in Figure 3 had the distributions of averages μ of the indexes for the EEGs groups with hypsarrhythmia and the EEGs with tracing without abnormalities. In these charts, it was observed that, on average, the indexes of the EEGs with hypsarrhythmia are bigger than the indexes with normal tracings.

From the data presented in the boxplots of Figure 3, we performed two hypothesis tests. In the first test (Kolmogorov–Smirnov), we verified the null hypothesis that the data comes from a normal pattern distribution. In this test, the result is 1 if the test rejects the null hypothesis in a significance level of 5%. The results of this test indicated the rejection of the null hypothesis in all datasets.

After we observed the results obtained through the Kolmogorov–Smirnov test, we decided to perform the Mann–Whitney test, in order to test the null hypothesis that the data were samples of continuous distributions with equal medians, against the alternative that they were not. The logical result 1 indicates a null hypothesis rejection and 0 indicates a failure to reject the null hypothesis at a significance level of 5%.

**Figure 4:**
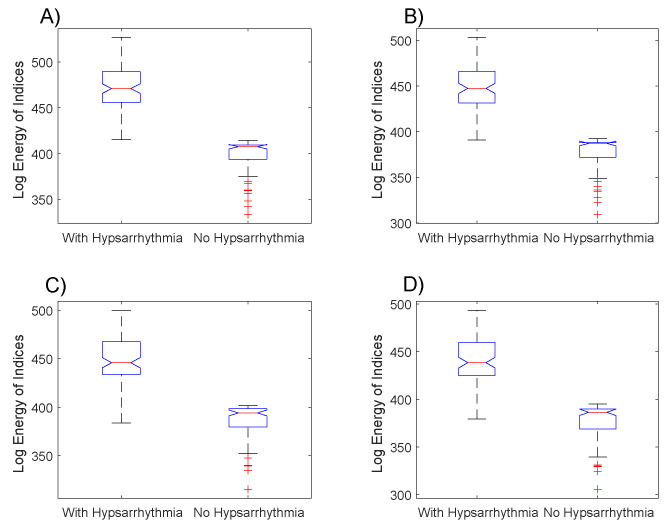
Boxplot with the log energy entropies of the EEGs’ indexes with and without hypsarrhythmia. Indexes generated from the Gabor function with the parameters: (**A**) $\sigma^{2}=20$, ω=1.02 and ϕ=5, (**B**) $\sigma^{2}=5$, ω=1.40 and ϕ=5, (**C**) $\sigma^{2}=15$, ω=2.68 and ϕ=5, (**D**) $\sigma^{2}=10$, ω=3.20 and ϕ=5.

The results obtained with the Mann-Whitney test indicated that the null hypothesis must be rejected in all the comparison situations presented in Figure 4, with the following values of *p*-value: $p=9.68\times 10^{-34}$ (3A), $p=9.67\times 10^{-34}$ (3B), $p=1.12\times 10^{-33}$ (3C) and $p=9.67\times 10^{-34}$ (3D).

The boxplot of Figure 4 shows the distribution of the log energy entropy $E_{LgEn}$ of the indexes for the EEGs groups with hypsarrhythmia and the EEGs with tracings without abnormalities. A large part of EEGs with a hypsarrhythmic pattern have log energy entropies of indexes bigger than those of the EEGs without abnormalities. This fact is similar to the results obtained with the μ averages.

**Figure 5:**
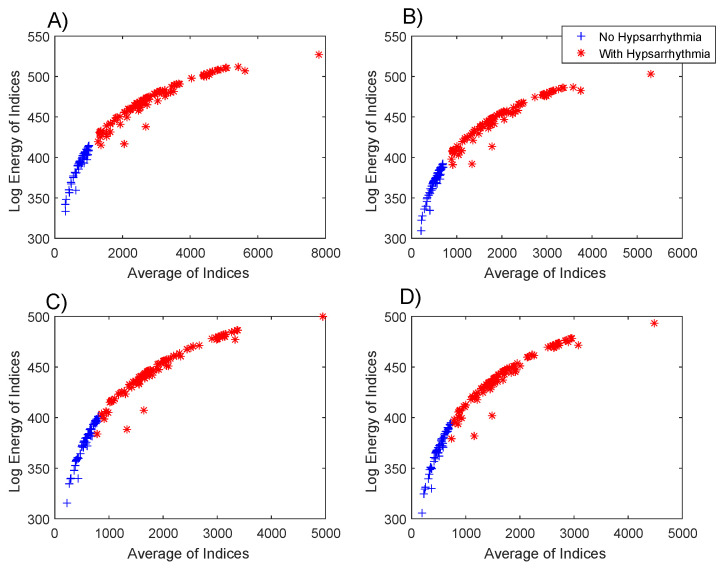
Dispersion of the average features and the log energy entropy of the EEGs with and without hypsarrhythmia. Features obtained from the Gabor function with the parameters: (**A**) $\sigma^{2}=20$, ω=1.02 and ϕ=5, (**B**) $\sigma^{2}=5$, ω=1.40 and ϕ=5, (**C**) $\sigma^{2}=15$, ω=2.68 and ϕ=5, (**D**) $\sigma^{2}=10$, ω=3.20 and ϕ=5.

The sequence of application of the hypothesis tests applied to the data presented in Figure 4 was repeated in the data of the log energy entropy. The results obtained with the Kolmogorov–Smirnov test were the same as those previously obtained. The results obtained with the Mann–Whitney test denote that the null hypothesis must be rejected in all comparison situations presented in Figure 5, with the following values of *p*-value: $p=9.77\times 10^{-34}$ (4A), $p=3.71\times 10^{-33}$ (4B), $p=4.84\times 10^{-32}$ (4C), $p=3.18\times 10^{-32}$ (4D).

Figure 5 shows the dispersion of the values of the average μ and the log energy entropy $E_{LgEn}$ of the indexes of each EEG, both for the group with hypsarrhythmia and for the group without abnormalities.

## 4. Discussion

In this paper, we proposed a methodology that aims to identify hypsarrhythmia patterns in EEGs. This methodology consisted of the mathematical elaboration of an index capable of highlighting significant differences between EEG signals with normal tracings and those with characteristics of hypsarrhythmia. In an initial analysis, it was possible to verify that there are important differences between the medians and the indexes’ entropies of the two groups under analysis, even when the parameters of the Gabor function are modified in Figure 3.

The results from the box plots of the indexes’ averages (Figure 3) and of the log energy entropy (Figure 4) were very similar, but the combination of these two metrics (Figure 5) resulted in a possible linear separation between the groups being analyzed.

The results presented in Figure 3 confirm the hypotheses suggested at the beginning of this paper that the index values of the electroencephalograms with hypsarrhythmia are, in general, bigger than the ones of encephalograms without any abnormalities. It is possible to observe, by comparing the boxplots of Figure 4A,B with the ones of Figure 4C,D, that the changes in the Gabor function’s parameters caused important implications for the results. The hypothesis tests applied showed that the proposed index is able to highlight the statistically significant differences, which enables this methodology to be used as a form of triage in the daily routine of the neuroscientists investigating epileptic syndromes in infants.

The proposed index can be easily implemented since it is based on the combination of mathematical operations already well-established in the literature, such as, for example, the median, the energy, and the DWT. This fact makes the computational calculation of the index rather quick.

It is relevant to emphasize that the parameters of the Gabor function chosen (based on the morphological characteristics of the spikes), highlight, in a mathematical way, one of the physiological features of the children with epilepsy. This feature consists of a large number of electric discharges in the shape of very peculiar waves (spikes).

## 5. Conclusions

Although it is important to discuss and elaborate on computational methods capable of performing the identification of hypsarrhythmia, it is also valid to emphasize that the existence of a concentration of kids (such as the ones assisted by the Casa Ninar) with this pathology is not common, and this can justify the lack of studies under this scope. However, the correct identification of hypsarrhythmia is an important step for the treatment of infant patients with refractory epilepsy, and the use of an index capable of assisting the electroencephalography expert is an important fact.

The findings confirm the hypotheses that the Gabor functions can enhance important differences between the groups in question. These results also suggest that the methodology of hypsarrhythmia’s identification based on an index is able to distinguish signals without abnormalities from signals with hypsarrhythmic characterístics.

The proposed index can be easily implemented and used as a screening factor, since it consists of a metric capable of summarizing in a single number (or in a sequence of indexes) the characteristics that indicate the presence or absence of hypsarrhythmia. It is important to stress that the proposed method aims only to assist the experts involved.
